# T2T genome, pan‐genome analysis, and heat stress response genes in *Rhododendron* species

**DOI:** 10.1002/imt2.70010

**Published:** 2025-03-05

**Authors:** Xiaojing Wang, Ping Zhou, Xiaoyu Hu, Yun Bai, Chenhao Zhang, Yanhong Fu, Ruirui Huang, Niu Suzhen, Xiaoming Song

**Affiliations:** ^1^ Institute of Agro‐Bioengineering/The Key Laboratory of Plant Resources Conservation and Germplasm Innovation in the Mountainous Region (Ministry of Education)/College of Life Sciences Guizhou University Guiyang China; ^2^ School of Life Sciences/School of Basic Medical Sciences North China University of Science and Technology Tangshan China; ^3^ Institute for Human Genetics University of California, San Francisco San Francisco California USA

## Abstract

This study reports the first high‐quality telomere‐to‐telomere (T2T) *Rhododendron liliiflorum* genome with 11 chromosomes that are gap free. The 24 telomeres and all 13 centromeres detected in this genome, which reached the highest quality gold level. In addition, other three *Rhododendron* species were sequenced and assembled to the chromosomal level. Based on 15 *Rhododendron* genomes, we conducted a pan‐genome analysis of genus *Rhododendron*. Combining the genome and whole transcriptome sequencing, we identified several key genes and miRNAs related to the heat stress, which were further verified by transgenic experiments. Our findings provide rich resources for comparative and functional genomics studies of *Rhododendron* species.

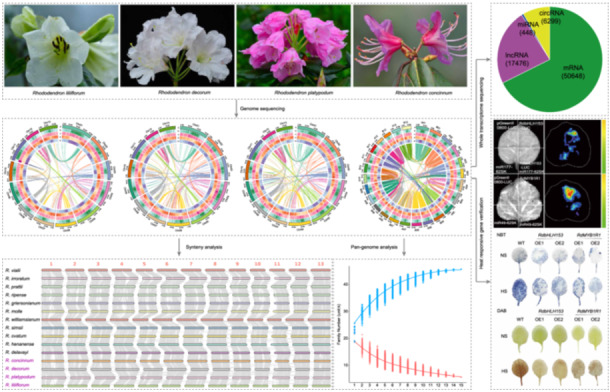

*Rhododendron* belongs to Ericaceae, which is one of the largest genus of woody plants. There are approximately 1000 *Rhododendron* species worldwide, and China is an important distribution center [1]. They underwent evolutionary radiations in Himalaya‐Hengduan Mountains, which are the world's biodiversity hotspots [2]. *Rhododendron* species are highly prized in horticulture due to their ornamental value.

Global climate change causes a rise in temperatures, while heat stress can influence the growth and development of plants [3, 4]. However, *Rhododendron* plants are typically adapted to cooler climates. Multi‐omics analysis and molecular techniques can be used to explore the heat stress response mechanism, which is of great significance for breeding heat‐tolerant varieties and expanding the *Rhododendron* cultivation range.

Although several genomic studies have been conducted separately on *Rhododendrons*, high‐quality telomere‐to‐telomere (T2T) genomes and large‐scale pan‐genome analysis of *Rhododendron* are still lacking, which limits our understanding of genetic diversity and gene mining [5, 6, 7, 8, 9, 10, 11, 12, 13, 14]. The T2T genome can provide more complete and comprehensive genomic information for a species [15]. Therefore, this study aims to resolve the first high‐quality T2T *Rhododendron* genome. Then, 15 *Rhododendron* genomes were used for pan‐genome analysis, identifying lots of structural variations (SVs), which provided rich resources for the mining of important functional genes and molecular breeding of *Rhododendron*.

## RESULTS AND DISCUSSION

### **Genome sequencing, assembly, and assessment of** *Rhododendron* **plants**

**Figure 1:**
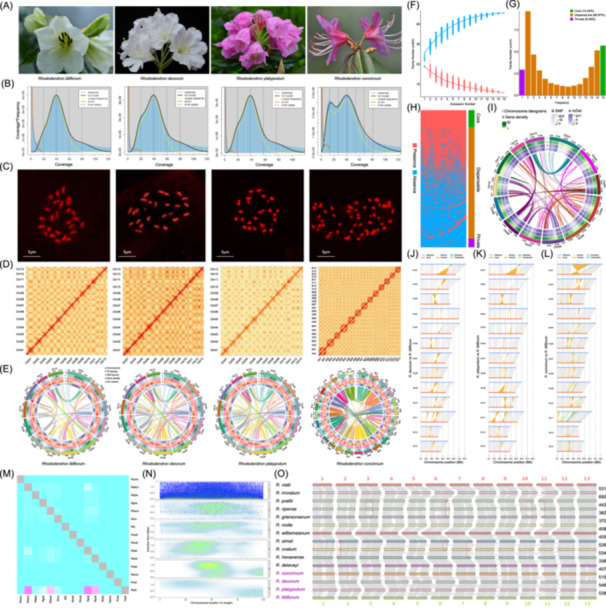
Genome analysis of four *Rhododendron* and other 11 published *Rhododendron* species. (A) Flowering photo of four *Rhododendron* species. (B) Genome survey by k‐mer. (C) Chromosome karyotype by flow cytometry. (D) Hi‐C contact map of genome assembly. (E) Distribution of transposable element, SSR, gene density, and GC content on chromosomes. (F) Trend chart of core (red) and noncore (blue) gene family quantities. (G) Family number of core, dispensable, and private clusters. (H) Presence and absence analysis of core, dispensable, and private clusters. (I) Distribution of gene density, SNP, and InDel variations across *R. liliiflorum* genome. (J–L) Homology and rearrangement of *R. decorum*, *R. platypodum*, and *R. concinnum* genome using T2T genome as reference. (M) Proportion of shared LTRs between two species (Rconc: *R. concinnum*; Rdeco: *R. decorum*; Rdela: *R. delavayi*; Rgrie: *R. griersonianum*; Rhena: *R. henanense*; Rirro: *R. irroratum*; Rlili: *R. liliiflorum*; Rmoll: *R. molle*; Rovat: *R. ovatum*; Rplat: *R. platypodum*; Rprat: *R. prattii*; Rripe: *R. ripense*; Rsims: *R. simsii*; Rvial: *R. vialii*; Rwill: *R. williamsianum*). (N) Distribution of LTRs on chromosomes. The x‐axis represents the percentage of chromosome positions, the *y*‐axis represents the insertion time of LTRs. (O) Genome synteny of 15 *Rhododendron* species. The number on the right indicated the syntenic block number.

Here, we perform the de novo genome sequencing of four *Rhododendron* plants (*Rhododendron liliiflorum*, *Rhododendron decorum*, *Rhododendron platypodum*, and *Rhododendron concinnum*) by PacBio HiFi, Oxford Nanopore Technology (ONT), Illumina, and Hi‐C technology (Figure 1A, Tables S1–6). The estimated genome size by K‐mer was 759.08, 581.05, 593.47, and 1356.22 Mb for *R. liliiflorum*, *R. decorum*, *R. platypodum*, and *R. concinnum*, respectively, which was further verified by flow‐cytometry (Table S1, Figure 1B).

We found that *R. concinnum* genome is almost twice as large as the other three species. Therefore, we further analyzed the chromosome karyotype using flow cytometry, for the first time, discovered that *R. concinnum* is a tetraploid with a karyotype of 2n = 4x = 52, which is distinctly different from the other three diploid species (2n = 2x = 26) (Figure 1C, Table S1).

The assembled genome size was 793.25, 649.87, 652.27, and 1321.11 Mb for four species (Table S1). The chromosomal anchored ratio was over 97.90% among four species by Hi‐C (Figure 1D, Table S5). We obtained four high‐quality assembled genomes with scaffold N50 over 48.68 Mb (Table S6). Core Eukaryotic Genes Mapping Approach value from 95.63% to 99.56%, Benchmarking Universal Single‐Copy Orthologs (BUSCO) value from 96.65% to 97.34%, and reads mapping rate exceeded 99.40% (Table S7).

Most importantly, we have obtained a high‐quality T2T *R. liliiflorum* genome, which consists of 13 chromosomes, with 24 telomeres and 13 centromeres detected (Figure S1A, Tables S8–S11). Eleven of the chromosomes are gap‐free from telomere to telomere, and the other two chromosomes only have one gap. The contig N50 of *R. liliiflorum* genome was over 58.56 Mb, which was larger than that of most previous Rhododendron genomes [1, 6, 7, 12]. The genome completeness is assessed by BUSCO (96.65%), and the genome consistency quality value (QV) is 43.71 (Table S1). Genome LTR assembly index (LAI) value is 21.15 (Figure S1B), indicating it has reached the highest quality gold level (LAI ≥ 20) [16].

### Genome annotation

Repetitive sequences accounted for over 49.10% of the four genomes, and most repetitive sequences were long‐terminal repeats (LTRs) (Figure 1E, Table S11). A total of 41,406, 41,084, 40,556, and 83,203 genes was predicted in the four genomes (Table S12). Over 97.15% of BUSCO genes were detected, indicating high completeness of prediction (Table S13). Over 92.16% of genes were annotated by NR, eggNOG, GO, KEGG, TrEMBL, KOG, Swissprot, and Pfam databases (Table S14). The 2355, 4862, 2852, and 9511 noncoding RNAs were detected in the four species (Table S15).

### **Pan‐genome analysis of 15** *Rhododendron* **genomes**

The genus Rhododendron, renowned for its diverse floral displays, has drawn significant scientific attention, with several genomes being decoded in recent years since the first *R. delavayi* genome was released [13]. Several Rhododendron genomes have been reported, such as *R. griersonianum* [11], *R. Henanense* [9], *R. Irroratum* [6], *R. kiyosumense* [10], *R. Ripense* [10], *R. Vialii* [8], *R. nivale* [5], and *R. williamsianum* [12]. These genomes are laying the groundwork for pan‐genome study [17, 18, 19, 20].

Based on these four high‐quality genomes, along with 11 previously published genomes, a pan‐genome analysis of *Rhododendron* genus was conducted (Figure 1F, Table S16). The T2T‐level *R. liliiflorum* genome was selected as the reference. This super‐pangenome has expanded the T2T‐level *R. liliiflorum* genome, by adding 394.57 Mb and 14,424 genes.

The number of gene families across 15 species is 45,731, including 5734 core gene families, 37,027 dispensable gene families, and 2970 private gene families (Figures 1G and S2A, Table S17). An UpSet plot was used to show the relationships of gene family sharing and uniqueness among 15 species. Finally, we constructed a distribution map of the presence and absence of gene families based on clustering analysis (Figure 1H).

Among 2970 private gene families, there were the most species‐specific genes in *R. irroratum* (1705) (Table S17, Figure S2B). The functional enrichment analysis indicated that “Sesquiterpenoid and triterpenoid biosynthesis” and “Linoleic acid metabolism” pathways were significantly enriched (Figure S3).

A total of 121,185 core genes were identified, and *R. ovatum* had the highest number (9847) (Figure S2B). Functional enrichment analysis indicated that gene pathways related to flower color and fragrance were significantly enriched, such as limonene and pinene degradation (Figure S4).

### **Variant analysis of 15** *Rhododendron* **genomes**

We perform a comprehensive identification of variations such as single nucleotide polymorphisms (SNPs), insertions and deletions (InDels), and SVs in *Rhododendron* based on pan‐genome analysis using T2T genome as reference (Figures 1I–L and S5).

The tetraploid *R. concinnum* had the highest number of SNPs (1,876,446) and InDels (447,281) (Figure 1I, Tables S18–19). Functional enrichment analysis showed that genes contained SNPs and InDels were significantly enriched in “Carbon metabolism” and “Biosynthesis of amino acids” pathways. *R. concinnum* had the highest number of SVs, reaching 7694 (Table S20). Meanwhile, we further subdivided SVs into duplication (DUP), translocation (TRANS), and inversion (INV), and found the former's quantity exceeded the latter two in most *Rhododendron* species (Figures S6–S7). Genes with SVs showed a distinct pattern compared to those with SNPs or InDels, focusing on RNA polymerase and mRNA surveillance pathway.

### **LTR analysis of 15** *Rhododendron* **genomes**

We identified 70,759 LTRs in the whole genome of 15 *Rhododendron* species, and *R. griersonianum* had the highest number of LTRs (7323) (Table S21). We found that most *Rhododendron* species only experienced one outbreak of insertion event during the last 1 million years (Mya), while *R. delavayi*, *R. molle*, and *R. williamsianum* experienced two outbreaks. Two events in *R. williamsianum* occurred at 1.53 and 2.94 Mya, earlier than other *Rhododendron* species.

We performed clustering on LTRs of 15 species to obtain the shared LTRs within each cluster. The results showed that 2622 LTRs could be clustered, with *R. platypodum* having the highest number (531). *R. liliiflorum* had the highest number of specific LTRs (109), while no species‐specific LTRs were found in *R. williamsianum*. The clustering diagram showed that *R. williamsianum* had the highest proportion of shared LTRs with other species (Figure 1M). Furthermore, the distribution density of LTRs in the middle of chromosomes was greater than two ends (Figure 1N).

### **Collinearity analysis of 15** *Rhododendron* **genomes**

Through collinearity analysis, we found that 15 *Rhododendron* genomes generally exhibit good collinearity (Figure 1O). The number of collinearity blocks from 336 (*R. henanense* vs. *R. delavayi*) to 692 (*R. irroratum* vs. *R. prattii*). Additionally, some genomic transpositions were detected, such as the terminal regions of chromosome 7 in *R. ovatum* compared to *R. simsii* and *R. henanense*.

### Whole transcriptome sequencing and detection of heat‐responsive gene

**Figure 2:**
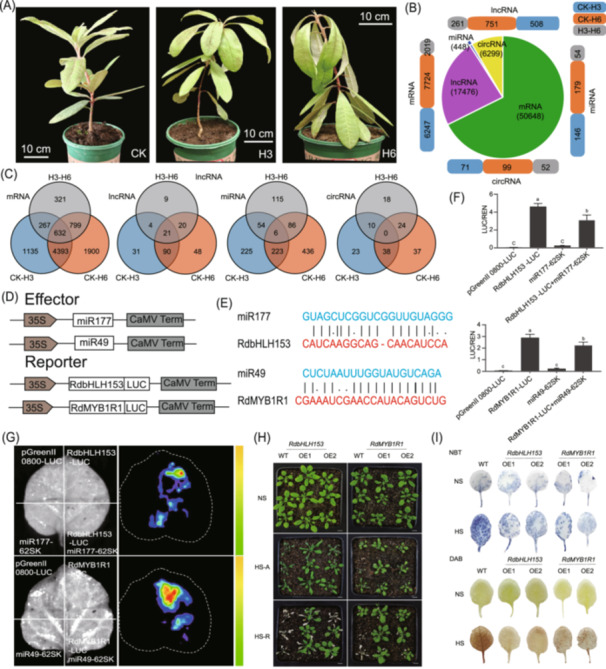
Whole transcriptome analysis and functional verification of miRNA and target genes. (A) The control (CK) and heat treatment (H3 and H6) of *R. delavayi*. (B) The identification and differently expressed analysis of mRNA, lncRNA, miRNA, and circRNA. (C) The specific and common differently expressed RNAs among three comparisons. (D) Diagram of effector and reporter constructions. (E) Schematic diagram of miR49 and miR177 target site mutation. (F) Luciferase assay statistics. Error bars indicate SEs from three replicates. Different lowercase letters indicate significant differences (*p* < 0.05). (G) Luciferase imaging assay. (H) Overexpression of *RdbHLH153* and *RdMYB1R1* enhanced heat tolerance. NS represent no heat stress, HS‐A represent heat stress for 36 h, and HS‐R represents that after 36 h of heat treatment, it is restored at normal temperature for 5 days. (I) Diaminobenzidine (DAB) and nitroblue tetrazolium (NBT) staining assay for detecting H_2_O_2_ and O^−2^ in transgenic lines and WT under no heat stress (NS) and heat stress (HS).

To explore heat‐resistant genes and regulatory mechanisms of *Rhododendrons*, we conducted whole transcriptome sequencing under heat treatment of CK, heat treatment of 3 days (H3) and 6 days (H6) (Figure 2A, Table S22). A total of 50,648 mRNAs, 17,476 lncRNAs, 448 miRNAs, and 6299 circRNAs were identified (Figure 2B). Furthermore, 632 mRNAs, 21 lncRNAs, and 6 miRNAs were differently expressed and shared among CK, H3, and H6 treatments (Figure 2C).

### Functional verification of candidate genes

We selected two representative pairs of miRNAs and related target genes for functional verification due to their response to heat treatment. Expression of the target genes was significantly upregulated at 3 and 6 h after heat treatment, while expression of the small RNAs was significantly downregulated. We further investigate the effects of miR177 on *RdbHLH153* (*Rhdel02G0118700*) expression and miR49 on RdMYB1R1 (*Rhdel08G0208700*) expression, respectively. Firefly luciferase was fused to the C‐terminal of RdbHLH153 and RdMYB1R1, respectively, and miR49 and miR177 were separately inserted into SK vectors (Figure 2D). Results showed that the target sites of miR177 in RdbHLH153 and miR49 in RdMYB1R1 were slightly altered (Figure 2E). The infiltrated areas within the single *Nicotiana benthamiana* leaves were infiltrated with mixtures of RdbHLH153/RdMYB1R1 and empty SK vector (mixed and infiltrated together), or the mixtures of RdbHLH153 and miR177 (RdMYB1R1 and miR49). All showed induction of luciferase signals, whereas the overexpressed *R. delavayi* miR177 and miR49 could abolish signals produced by RdbHLH153/RdMYB1R1 (Figure 2E–G).

To further investigate the roles of *RdbHLH153* and *RdMYB1R1* in heat stress, transgenic *Arabidopsis* lines overexpressing *RdbHLH153* and *RdMYB1R1* were generated using floral‐dip method (Table S23). After 36 h heat treatment, the growth of transgenic plants was significantly better than WT (Figure 2H). After heat treatment, the seedlings were returned to normal conditions for 5 days, and it was found that transgenic plants turn alive, while all leaves of WT became yellowish. This result indicated that *RdbHLH153* and *RdMYB1R1* play important roles in enhancing heat tolerance. DAB and NBT staining revealed that the contents of H_2_O_2_ and O^−2^ in *RdbHLH153/RdMYB1R1*‐OE lines were significantly decreased comparing with WT plants (Figure 2I).

## DISCUSSION

The genus *Rhododendron*, renowned for its diverse floral displays, has drawn significant scientific attention, with several genomes being decoded in recent years since the first *R. delavayi* genome was released [13]. Through genome sequencing of nine *Rhododendron* species, researchers have revealed the molecular mechanisms underlying the formation of flower color diversity [1]. In addition, several *Rhododendron* genomes have been reported, such as *R. griersonianum* [11], *R. Henanense* [9], *R. Irroratum* [6], *R. Kiyosumense* [10], *R. Ripense* [10], *R. Vialii* [8], *R. nivale* [5], and *R. williamsianum* [12]. These genomes and related database are laying the groundwork for a more comprehensive understanding of the comparative and functional genomics study [17, 18, 19, 20].

Although several genomes have been sequenced for *Rhododendron*, none of them reached the T2T level, especially for the large‐scale pan‐genome analysis of *Rhododendron* using a T2T genome as reference. Here, *R. liliiflorum* genome was deciphered at the T2T level. Compared to the genomes previously released, we obtained a higher quality and more complete genome for *Rhododendron*. The contig N50 of *R. liliiflorum* genome was over 58.56 Mb, which was larger than that of most previous *Rhododendron* genomes [1, 6, 7, 12]. Furthermore, large‐scale pan‐genome analysis of 15 *Rhododendron* genomes identified lots of SVs, which is used for understanding the genetic diversity behind the various morphotypes.

## CONCLUSION

In conclusion, we report the first high‐quality T2T *R. liliiflorum* genome. Genome sequencing of three other *Rhododendron* species was completed, and *R. concinnum* was discovered to be tetraploid. Pan‐genome analysis of 15 *Rhododendron* genomes detected structure variations and identified several key heat‐stress‐related genes.

## METHODS

The principal categories of methods employed in this study are presented as follows. The detailed descriptions of these methods can be found in the supplementary text.

(a) Genome sequencing, genome size estimation, and chromosome karyotype analysis
(b) Data quality control and *de novo* genome assembly
(c) Hi‐C data processing and assisted genome assembly
(d) Telomere‐to‐Telomere (T2T) genome analysis
(e) Genomic repetitive sequence annotation
(f) Gene prediction, evaluation, and functional annotation
(g) Noncoding RNA prediction
(h) RNA extraction
(i) Graph‐based genome construction
(j) Core and noncore gene family analysis
(k) Variant analysis
(l) Long‐terminal repeats (LTR) insertion time analysis
(m) Genome collinearity and visualization
(n) Plant materials and treatment
(o) Library construction and whole transcriptome sequencing
(p) Analysis of mRNAs and noncoding RNAs
(q) Dual‐luciferase transient expression system
(r) Generation of transgenic plant materials and heat stress treatment
(s) Detection of reactive oxygen species (ROS).

## AUTHOR CONTRIBUTIONS

**Xiaojing Wang**: Conceptualization; data curation; investigation; funding acquisition; supervision; writing—original draft; project administration; writing—review and editing; resources; validation; visualization; formal analysis. **Ping Zhou**: Validation; data curation; writing—original draft; formal analysis; visualization; writing—review and editing. **Xiaoyu Hu**: Data curation; validation; formal analysis; visualization; writing—original draft; writing—review and editing. **Yun Bai**: Data curation; formal analysis; visualization; validation; writing—original draft. **Chenhao Zhang**: Data curation; formal analysis; visualization; validation; writing—original draft; methodology. **Yanhong Fu**: Formal analysis; visualization; validation; writing—original draft. **Ruirui Huang**: Writing—review and editing; supervision; data curation. **Niu Suzhen**: Investigation; supervision; writing—original draft; writing—review and editing; resources; validation; data curation. **Xiaoming Song**: Conceptualization; data curation; formal analysis; visualization; writing—original draft; writing—review and editing; project administration; supervision; investigation; validation; funding acquisition; resources; methodology; software.

## CONFLICT OF INTEREST STATEMENT

The authors declare no conflict of interest.

## ETHICS STATEMENT

No animals or humans were involved in this study.

## Supporting information


**Figure S1:** The assessment of Telomere‐to‐Telomere (T2T) genome of *R. liliiflorum*.
**Figure S2:** The gene family analysis of 15 species.
**Figure S3:** The KEGG functional enrichment analysis on all species‐specific genes in *R. liliiflorum*.
**Figure S4:** The KEGG functional enrichment analysis on all core cluster genes of 15 *Rhododendron* genomes.
**Figure S5:** The comparative genome visualization map shows the homology and rearrangement between each *Rhododendron* species and the reference T2T genome of *R. liliiflorum*.
**Figure S6:** The length distribution of three structural variations (SVs) types, including duplication (DUP), inversion (INV), and translocation (TRANS) in the *Rhododendron* genome.
**Figure S7:** The length of duplication (DUP), translocation (TRANS), and inversion (INV) type of structural variations (SVs) in each *Rhododendron* species.

**Table S1:** Statistics of *Rhododendron* genome sequencing, assembly and annotation.
**Table S2:** Statistics of sequencing data obtained by illumina Hiseq platform for genome survey of four *Rhododendron* species.
**Table S3:** Statistics of sequencing data of four *Rhododendron* species by Pacbio HiFi platform.
**Table S4:** Statistics of sequencing data of *Rhododendron liliiflorum* by ONT platform.
**Table S5:** The assembled length and cluster number of each chromosome of *Rhododendron liliiflorum* genome by HIC.
**Table S6:** Statistics of sequencing data of four *Rhododendron* species by HIC platform.
**Table S7:** CEGMA and BUSCO assessment of assembled genome by Pacbio HiFi platform.
**Table S8:** The assembled chromosome length and gap informatioin of *Rhododendron liliiflorum* T2T genome.
**Table S9:** The telomere position of assembled *Rhododendron liliiflorum* T2T genome.
**Table S10:** The centromere position of assembled *Rhododendron liliiflorum* T2T genome.
**Table S11:** The statistics of the repeat sequence classification in four *Rhododendron* genomes.
**Table S12:** The statistics of predicted gene number in the four assembled *Rhododendron* genomes.
**Table S13:** BUSCO assessment of genes in the four *Rhododendron* genomes.
**Table S14:** Statistics of gene functional annotations in the four *Rhododendron* genomes.
**Table S15:** The ncRNA number of the four *Rhododendron* genomes.
**Table S16:** Statistics of gene family in the 15 *Rhododendron* genomes by pan‐genome analysis.
**Table S17:** Statistics of core, dispensable, and private number in the 15 *Rhododendron* genomes by pan‐genome analysis.
**Table S18:** Statistics of SNP number in the 15 *Rhododendron* genomes.
**Table S19:** Statistics of INDEL number in the 15 *Rhododendron* genomes.
**Table S20:** Statistics of SV number in the 15 *Rhododendron* genomes.
**Table S21:** Statistics of LTR cluster in the 15 *Rhododendron* genomes.
**Table S22:** Whole transcriptome sequencing data assessment of *Rhododendron* species under heat treatment.
**Table S23:** Transgenic and dual luciferase assay primers used in this study.

## Data Availability

All the sequencing data have been deposited in NCBI under submission number SUB15033098, BioProject accession number PRJNA1215314 (https://www.ncbi.nlm.nih.gov/sra/PRJNA1215314). The data and scripts used are saved in GitHub (https://github.com/songxm-ncst/Rhododendron). All the genomic annotation datasets have also been curated in the download interface of TEGR database (http://www.tegr.com.cn) with species latin name. Supplementary materials (methods, figures, tables, graphical abstract, slides, videos, Chinese translated version and update materials) may be found in the online DOI or iMeta Science http://www.imeta.science/.

## References

[1] Xia, Xiaomei , Huilong Du , Xiaodi Hu , Jingjie Wu , Fusheng Yang , Congli Li , Sixin Huang , Qiang Wang , Chengzhi Liang , and Xiaoquan Wang . 2024. “Genomic Insights into Adaptive Evolution of the Species‐Rich Cosmopolitan Plant Genus Rhododendron.” Cell Reports 43: 114745. 10.1016/j.celrep.2024.114745 39298317

[2] Ma, Yazhen , Xingxing Mao , Ji Wang , Lei Zhang , Yuanzhong Jiang , Yuying Geng , Tao Ma , et al. 2022. “Pervasive Hybridization During Evolutionary Radiation of Rhododendron Subgenus Hymenanthes in Mountains of Southwest China.” National Science Review 9: nwac276. 10.1093/nsr/nwac276 36687562 PMC9844246

[3] Shen, Huifei , Bing Zhao , Jingjing Xu , Wen Liang , Wenmei Huang , and Houhua Li . 2017. “Effects of Heat Stress on Changes in Physiology and Anatomy in Two Cultivars of Rhododendron.” South African Journal of Botany 112: 338–345. 10.1016/j.sajb.2017.06.018

[4] Wang, Yujie , Shuqi Sun , Xuehuan Feng , Nan Li , and Xiaoming Song . 2024. “Two lncRNAs of Chinese Cabbage Confer Arabidopsis With Heat and Drought Tolerance.” Vegetable Research 4: e029. 10.48130/vegres-0024-0029

[5] Lyu, Zhenyu , Xiongli Zhou , Siqi Wang , Gaoming Yang , Wenguang Sun , Jieyu Zhang , Rui Zhang , and Shikang Shen . 2024. “The First High‐Altitude Autotetraploid Haplotype‐Resolved Genome Assembled (Rhododendron Nivale Subsp. Boreale) Provides New Insights Into Mountaintop Adaptation.” GigaScience 13: giae052. 10.1093/gigascience/giae052 39110622 PMC11304948

[6] Wu, Xiaopei , Lu Zhang , Xiuyun Wang , Rengang Zhang , Guihua Jin , Yanting Hu , and Hong Yang , et al. 2023. “Evolutionary History of Two Evergreen Rhododendron Species as Revealed by Chromosome‐Level Genome Assembly.” Frontiers in Plant Science 14: 1123707. 10.3389/fpls.2023.1123707 37025132 PMC10070854

[7] Nie, Shuai , Shiwei Zhao , Tianle Shi , Wei Zhao , Rengang Zhang , Xuechan Tian , and Jingfang Guo , et al. 2022. “Gapless Genome Assembly of Azalea and Multi‐Omics Investigation into Divergence Between Two Species With Distinct Flower Color.” Horticulture Research 10: uhac241. 10.1093/hr/uhac241 36643737 PMC9832866

[8] Chang, Yuhang , Rengang Zhang , Yongpeng Ma , and Weibang Sun . 2023. “A Haplotype‐Resolved Genome Assembly of Rhododendron Vialii Based on PacBio HiFi Reads and Hi‐C Data.” Scientific Data 10: 451. 10.1038/s41597-023-02362-1 37438373 PMC10338486

[9] Zhou, Xiao‐Jun , Jian‐Tao Li , Hai‐Liang Wang , Jun‐Wang Han , Kai Zhang , Shuai‐Wei Dong , Yan‐Zhao Zhang , Hui‐Yuan Ya , Yan‐Wei Cheng , and Shan‐Shan Sun . 2022. “The Chromosome‐Scale Genome Assembly, Annotation and Evolution of Rhododendron Henanense Subsp. Lingbaoense.” Molecular Ecology Resources 22: 988–1001. 10.1111/1755-0998.13529 34652864

[10] Shirasawa, Kenta , Nobuo Kobayashi , Akira Nakatsuka , Hideya Ohta , and Sachiko Isobe . 2021. “Whole‐Genome Sequencing and Analysis of Two Azaleas, Rhododendron Ripense and Rhododendron Kiyosumense.” DNA Research 28: dsab010. 10.1093/dnares/dsab010 34289022 PMC8435550

[11] Ma, Hong , Yongbo Liu , Detuan Liu , Weibang Sun , Xiongfang Liu , Youming Wan , Xiujiao Zhang , et al. 2021. “Chromosome‐Level Genome Assembly and Population Genetic Analysis of a Critically Endangered Rhododendron Provide Insights into Its Conservation.” The Plant Journal 107: 1533–1545. 10.1111/tpj.15399 34189793

[12] Soza, Valerie L. , Dale Lindsley , Adam Waalkes , Elizabeth Ramage , Rupali P. Patwardhan , Joshua N. Burton , Andrew Adey , et al. 2019. The Rhododendron Genome and Chromosomal Organization Provide Insight Into Shared Whole‐Genome Duplications Across the Heath Family (Ericaceae).” Genome Biology and Evolution 11: 3353–3371. 10.1093/gbe/evz245 31702783 PMC6907397

[13] Zhang, Lu , Pengwei Xu , Yanfei Cai , Lulin Ma , Shifeng Li , Shufa Li , and Weijia Xie , et al. 2017. “The Draft Genome Assembly of Rhododendron Delavayi Franch. Var. Delavayi.” GigaScience 6: gix076. 10.1093/gigascience/gix076 PMC5632301 29020749

[14] Wen, Sulin , Xiaowei Cai , Kun Yang , Yi Hong , Fuhua Fan , Qian Wang , and Bingxue Zhang , et al. 2024. “Chromosome‐Level Genome Assembly of a Rare Karst‐Growing Rhododendron Species Provides Insights into Its Evolution and Environmental Adaptation.” Journal of Systematics and Evolution 1–23. 10.1111/jse.13130

[15] Wei, Chuanzheng , Lei Gao , Ruixue Xiao , Yanbo Wang , Bingru Chen , Wenhui Zou , Jihong Li , Emma Mace , David Jordan , and Yongfu Tao . 2024. “Complete Telomere‐to‐Telomere Assemblies of Two Sorghum Genomes to Guide Biological Discovery.” iMeta 3: e193. 10.1002/imt2.193 38882488 PMC11170960

[16] Ou, Shujun , Jinfeng Chen , and Ning Jiang . 2018. “Assessing Genome Assembly Quality Using the LTR Assembly Index (LAI).” Nucleic Acids Research 46: e126. 10.1093/nar/gky730 30107434 PMC6265445

[17] Wang, Xiaojing , Yunfeng Wei , Zhuo Liu , Tong Yu , Yanhong Fu , and Xiaoming Song . 2023. “TEGR: A Comprehensive Ericaceae Genome Resource Database1.” Journal of Integrative Agriculture 24, no. 3: 1140–1151. 10.1016/j.jia.2023.11.026

[18] Feng, Shuyan , Zhuo Liu , Huilong Chen , Nan Li , Tong Yu , Rong Zhou , Fulei Nie , Di Guo , Xiao Ma , and Xiaoming Song . 2024. “PHGD: An Integrative and User‐Friendly Database for Plant Hormone‐Related Genes.” iMeta 3: e164. 10.1002/imt2.164 38868516 PMC10989150

[19] Liu, Zhuo , Chenhao Zhang , Jinghua He , Chunjin Li , Yanhong Fu , Yongfeng Zhou , Rui Cao , Haibin Liu , and Xiaoming Song . 2024. “plantGIR: A Genomic Database of Plants.” Horticulture Research 11: uhae342. 10.1093/hr/uhae342 39712867 PMC11661351

[20] Wu, Tong , Zhuo Liu , Tong Yu , Rong Zhou , Qihang Yang , Rui Cao , Fulei Nie , Xiao Ma , Yun Bai , and Xiaoming Song . 2024. “Flowering Genes Identification, Network Analysis, and Database Construction for 837 Plants.” Horticulture Research 11: uhae013. 10.1093/hr/uhae013 38585015 PMC10995624

