# Supplementary material for: T2T genome, pan‐genome analysis, and heat stress response genes in Rhododendron species
Source: Imeta. 2025 Mar 5;4(2):e70010. doi: 10.1002/imt2.70010 (PMC11995181; doi:10.1002/imt2.70010)
Supplement: Supplementary file 1 — Figure S1: The assessment of Telomere‐to‐Telomere (T2T) genome of R. liliiflorum. Figure S2: The gene family analysis of 15 species. Figure S3: The KEGG functional enrichment analysis on all species‐specific genes in R. liliiflorum. Figure S4: The KEGG functional enrichment analysis on all core cluster genes of 15 Rhododendron genomes. Figure S5: The comparative genome visualization map shows the homology and rearrangement between each Rhododendron species and the reference T2T genome of R. liliiflorum. Figure S6: The length distribution of three structural variations (SVs) types, including duplication (DUP), inversion (INV), and translocation (TRANS) in the Rhododendron genome. Figure S7: The length of duplication (DUP), translocation (TRANS), and inversion (INV) type of structural variations (SVs) in each Rhododendron species. [file IMT2-4-e70010-s002.docx]

**Supporting information to:**

**T2T genome, pan-genome analysis, and heat stress response genes in *Rhododendron* species**

**Running title:** T2T genome and pan-genome analysis of *Rhododendron*

Xiaojing Wang^1^^#^, Ping Zhou^1#^, Xiaoyu Hu^1#^, Yun Bai^2#^, Chenhao Zhang^2^^#^, Yanhong Fu^2^, Ruirui Huang^3^, Suzhen, Niu^1*^, Xiaoming Song^2*^

^1^Institute of Agro-Bioengineering/The Key Laboratory of Plant Resources Conservation and Germplasm Innovation in the Mountainous Region (Ministry of Education)/College of Life Sciences, Guizhou University, Guiyang 550025, China

^2^School of Life Sciences/School of Basic Medical Sciences, North China University of Science and Technology, Tangshan 063210, China

^3^Institute for Human Genetics, University of California, San Francisco, San Francisco, CA 94143, USA

^#^ The authors contributed equally: Xiaojing Wang, Ping Zhou, Xiaoyu Hu, Yun Bai, Chenhao Zhang

*Correspondence: [songxm@ncst.edu.cn](mailto:songxm@ncst.edu.cn) (Xiaoming Song); [niusuzhen@163.com](mailto:niusuzhen@163.com) (Suzhen Niu)

**Materials and Methods**

**Genome sequencing, genome size estimation, and chromosome karyotype analysis**

The DNA was extracted from four *Rhododendron* plant leaves (*R*. *liliiflorum*, *R.* *decorum*, *R*. *platypodum*, and *R. concinnum*) using QIAGEN kit. DNA purity and quantification was determined by NanoDrop™ One spectrophotometer and Qubit^®^ 3.0 Fluorometer, respectively. Sequencing libraries were constructed and sequenced using the Illumina and PacBio-HiFi platforms according to previous reports [1, 2]. Furthermore, Hi-C technology was used to assist genome assembly to the chromosomal level. In addition, the Oxford Nanopore Technology (ONT) Super-long read sequencing libraries were constructed and sequenced for *R. liliiflorum* according to the manufacturer’s protocol. The genome size was estimated according to the K-mers using Illumina sequencing data [3]. In addition, the flow cytometry method was used to estimate the genome size and chromosome karyotype.

**Data quality control and *de novo* genome assembly**

The quality control of PacBio-HiFi data was performed by SMRT Link program (https://www.pacb.com/support/software-downloads/). Based on the subreads, ccs software (https://github.com/PacificBiosciences/ccs) was used to obtain high-precision HiFi reads (min-passes = 3 and min-rq = 0.99). The genome assembly was performed by the Hifiasm program (v0.19), which was developed according to the characteristics of PacBio HiFi [4]. Hifiasm assembly mainly contained three steps, including error correction, construction of assembly graph, and generation of assembly sequence.

**Hi-C data processing and assisted genome assembly**

Filter the raw data from Hi-C sequencing to remove adapter sequences and low-quality reads to obtain high-quality clean data. Use HiC-Pro (version 2.10.0) to filter and assess the Hi-C data [5]. By analyzing the alignment results, identify valid and invalid interaction pairs in the Hi-C sequencing results, thus achieving quality assessment of the Hi-C library. Use bwa (version: 0.7.17-r1188) to align the clean data with the preliminarily assembled genome, obtaining mapped data [6]. Then, the ALLHiC program was used to assist the four *Rhododendron* plants genome assembly [7]. Clustered bam files and genomes from ALLHiC were showed by the Juicebox program [8]. Finally, the assembled genome was assessed by BUSCO (v5.8.2) and CEGMA (v2.0) software [9, 10].

**Telomere-to-Telomere (T2T) genome analysis**

The quarTeT software (<https://github.com/aaranyue/quarTeT>) uses contig-based genomes to fill gaps in chromosomal genomes, ultimately obtaining a T2T genome. The software Merqury (<https://github.com/marbl/merqury>) is used to compare high-accuracy sequencing Reads k-mers with genome assemblies for assessment, obtaining consistency quality (QV). LTR assembly index (LAI) is used to evaluate genome integrity [11]. The TIDK (<https://github.com/tolkit/telomeric-identifier>) software retrieves telomeric repeat units in the genome, and FindTelomeres (<https://github.com/JanaSperschneider/FindTelomeres>) uses these repeat units to identify potential telomeric sequences and the location of telomeres. The Centromics (<https://github.com/ShuaiNIEgithub/Centromics>) software uses third-generation reads to obtain potential centromeric repeats and then maps them back to the genome to obtain the location and sequence of centromeres. The R package RIdeogram is used to draw chromosome maps, marking gaps, telomeres, and centromeres [12].

**Genomic repetitive sequence annotation**

We first use RepeatModeler2 (version 2.0.1) for de novo prediction [13], which mainly calls two de novo prediction softwares: RECON (version 1.0.8) and RepeatScout (version 1.0.6) [14, 15]. We also use RepeatClassifier with the help of the known database Dfam (version 3.5) to classify the predicted results. Secondly, we use LTR_retriever [16] (version 2.9.0) specifically for de novo prediction of LTRs, which mainly relies on the prediction results of LTRharvest [17] (version 1.5.10) and LTR_FINDER [18] (version 1.07). Then, the above de novo prediction results are merged with the known databases to eliminate redundancy and obtain a species-specific repetitive sequence database. Finally, RepeatMasker [19] (version 4.1.2) is used to predict transposable element (TE) sequences in the genome based on the constructed repetitive sequence database.

**Gene prediction, evaluation, and functional annotation**

Gene prediction and assessment are conducted using three methods: homology-based prediction, de novo prediction, and transcriptome-based prediction. Specifically, de novo prediction is performed using Augustus [20] (version 3.1.0) and SNAP [21]. Homology-based prediction is carried out using GeMoMa [22] (version 1.7). Transcriptome-based prediction primarily involves two approaches to assemble transcripts for prediction. One approach uses Hisat [23] (version 2.1.0) and Stringtie [24] (version 2.1.4) to obtain transcripts and employs GeneMarkS-T [25] (version 5.1) for gene prediction. Another approach involves assembling transcripts through Trinity [26] (version 2.11) and then using PASA [27] (version 2.4.1) for gene prediction. Finally, the predictions from the three methods are integrated using EVM [28] (version 1.1.1), and PASA is used for refinement. The BUSCO (v5.2.2) software was used to assess the completeness of gene prediction [10]. Annotating and analyzing the predicted gene sequences using databases such as NR, eggnog [29], GO, KEGG [30], TrEMBL [31], KOG, SWISS-PROT [31], and Pfam [32].

**Non-coding RNA prediction**

Non-coding RNA mainly includes various types of RNAs, such as microRNA, rRNA, and tRNA. tRNA is identified using tRNAscan-SE [33] (version 1.3.1). rRNA prediction primarily uses barrnap (https://github.com/tseemann/barrnap) (version 0.9). miRNA, snoRNA, and snRNA are predicted based on the Rfam [34] (version 14.5) database and using Infernal [35] (version 1.1).

**RNA extraction**

Total RNA was isolated using RNAiso Plus (TaKaRa Bio, Japan). Integrity and quality were assessed by electrophoresis and spectrophotometry. First-strand cDNA was synthesized using the PrimeScript RT Reagent Kit, according to the manufacturer’s instructions.

**Graph-based genome construction**

Using the genome of 15 *Rhododendron* genomes in this study, a graphical pangenome was created via minigraph [36]. The resulting pangenome was converted using gfatools (<https://github.com/lh3/gfatools>).

**Core and non-core gene family analysis**

Using the Orthofinder v2.4.0 software to classify protein sequences from 15 species into gene families (E-value < 0.001), and then annotate the obtained gene families using the PANTHER V15 database [37]. Subsequently, perform GO and KEGG enrichment analysis on Core, Dispensable, and Private gene families using the clusterProfiler v3.14.0 [38].

**Variant analysis**

Using the *Rhododendron liliiflorum* genome as a reference, whole-genome alignment is performed using MUMmer 4.0 [39]. Subsequently, variants are detected using SyRI, which can identify regions of synteny, structural rearrangements (inversions, translocations, and duplications), and local variations (SNPs, indels, CNVs) [40]. The ANNOVAR software toolkit is then used to annotate the regions in the genome where the variants occur [41]. By comparing with the reference genome, the density of SNP and InDel variant sites in all samples is statistically analyzed and displayed using a circos plot.

**Long-terminal repeats (LTR) insertion time analysis**

Utilizing the LTR_FINDER v1.07 software to identify LTR sequences in the genome with the parameter "-S 6" [18], while also filtering out duplicate results from LTR_FINDER. Extract the flanking sequences on both sides of the LTRs, perform alignment with MAFFT (--localpair --maxiterate 1000) [42]. Distances are calculated using the Kimura model in the EMBOSS v6.6.0 software [43]. The formula for calculating time is *T* = K / (2 × *r*), where the molecular clock rate *r* is taken as 7 × 10^-9 [44]. Clustering of LTRs from all species is performed using dbcluster from vmatch, to obtain shared LTRs within each cluster (<https://github.com/genometools/vstree>).

**Genome collinearity and visualization**

First, perform alignment using BLAST with an E-value threshold of less than 1e-5. Then, use MCScanX to identify collinear regions in the genome, and synteny plot among different species are visualized using the MCScan in Python version [45].

**Plant materials and treatment**

*R. delavayi* plants grown for three years at the experimental field of Guizhou university were selected as experiment material. The 3-year-old *R. delavayi* seedling were transferred into growth chambers set at 20 ℃ to 25 ℃ with 80% relative humidity for one month. All plants were separated into control group (25 ℃, CK) and high-heat stress (38 ℃) at 3 day (H3) and 6 day (H6), and each group contained three biological replicates. The first and second leaves of each group were sampled, immediately frozen in liquid nitrogen, and stored at -80 ℃ until used for whole transcriptome RNA-seq.

**Library construction and whole transcriptome sequencing**

The total RNA from leaf samples was extracted and purified using Trizol reagent (Invitrogen, CA, USA). The RNA content and quality were evaluated with a NanoDrop ND-1000 spectrophotometer (Wilmington, DE, USA) and an Agilent 2100 Bioanalyzer (CA, USA). Each leaf sample group (CK, H3, and H6) included three biological replicates, resulting in a total of nine libraries. These qualified libraries were sequenced using the Illumina platform for comprehensive transcriptome analysis, encompassing mRNAs, lncRNAs, and circRNAs. Additionally, nine small RNA libraries were prepared using the TruSeq Small RNA Sample Prep Kits (Illumina, San Diego, USA) to profile miRNA expression. These libraries were sequenced on the Illumina NovaSeq 6000 platform.

**Analysis of mRNAs and non-coding RNAs**

The raw sequencing reads were processed with Cutadapt software to eliminate reads containing undetermined bases, adapters, or low-quality sequencing data [46]. The quality of the data was confirmed using the FastQC tool (<https://github.com/s-andrews/FastQC>). Valid data was aligned to the reference genome of *R. delavayi*. Expression levels were determined using the StringTie and edgeR programs [24, 47]. For lncRNA prediction, the Coding Potential Calculator (CPC) and Coding-Non-Coding Index (CNCI) were employed [48, 49]. CircRNAs were identified using the find_circ tool [50]. Clean reads were mapped against the Silva, GtRNAdb, Rfam, miRBase, and Repbase databases to identify miRNAs. Differentially expressed mRNAs (DEmRNAs), lncRNAs (DElncRNAs), circRNAs (DEcircRNAs), and miRNAs (DEmiRNAs) were detected using DESeq2 with the threshold criteria of |log2 (fold-change)| ≥ 1 and *p* < 0.05 [51]. Target genes of miRNAs and circRNAs were predicted using TargetFinder [52].

**Dual-luciferase transient expression system**

The dual-luciferase reporter system was applied to investigate the interaction between miRNA and target genes [53]. The precursors of miR_49 and miR_177 were cloned into pGreenII 62SK vector [54], and full length coding sequences (CDSs) of *RdbHLH153* (*Rhdel02G0118700*) and *RdMYB1R1* (*Rhdel08G0208700*) were inserted into pGreenII 0800-LUC vector [54]. All plasmid DNA were reintroduced into *A. tumefaciens* (GV3101). The *A. tumefaciens* strains were cultured with Luria-Bertani (LB) medium (10 g tryptone, 5 g yeast extract, and 10 g NaCl), OD_600_ to 1. For dual-luciferase assays, the ratio of *A. tumefaciens* mixtures of 62SK and the corresponding 0800-LUC was 10: 1, which were then infiltrated into the leaves of 4-week-old *N. benthamiana*. Three days after injection, firefly luciferase and Renilla luciferase were detected using a Promega GloMax96 instrument and the Dual-Luciferase Reporter Assay System (Promega). Dual-luciferase analyses were conducted with three biological replicates, and all primers used for SK and LUC vector construction are listed in Table S23.

**Generation of transgenic plant materials and heat stress treatment**

To verify the function of RdbHLH153 and RdMYB1R1 in response to heat stress, the full-length CDSs of two genes were cloned into the pCAMBIA2301 vector derived by CaMV35s promoter to generate the constructs 35S::RdbHLH153-2301, 35S::RdMYB1R1-2301. The primer sequences used in this research were shown in Table S23. The constructed vectors were separately introduced into *A. tumefaciens* (GV3101), which were applied to transform *Arabidopsis* using the floral-dip method [55]. Two independent T3 transgenic *Arabidopsis* lines were used for heat tolerance analyses.

The seeds of T3 Transgenic *Arabidopsis* lines overexpressing *RdbHLH153*, *RdMYB1R1* and WT were sown on matrix soil-vermiculite (2:1, v/v), and placed in a growth chamber with 70% humidity and 16-h light/8-h dark cycle at 22 °C/20 °C for 20 days. The four-week-old *Arabidopsis* were exposed to selected for heat treatment. For the DAB and NBT staining, the plants were exposed to 45 °C for heat treatment for 36 h, and then transferred into normal condition.

**Detection of reactive oxygen species (ROS)**

Mature leaves of 4-week-old WT and transgenic *Arabidopsis* lines were applied for DAB and NBT staining as previously described [56]. The DAB was dissolved in sterilized water and adjusted to pH 3.8 with KOH used for H_2_O_2_ staining. Four-week-old WT and *Arabidopsis*-OX lines were vacuum infiltrated in 1.25 mg/mL DAB solution, and then cleared in boiling 95% (v/v) ethanol for 10 min. For NBT staining, mature leaves were immersed and infiltrated under vacuum with 3.5 mg/mL NBT (N6876, Sigma-Aldrich) staining solution in 10 mM potassium phosphate buffer containing 10 mM sodium azide. After vacuum infiltration, stained leaves were bleached in boiling 95% ethanol (v/v) for 10 min.

**References**

1. Song, Xiaoming, Haibin Liu, Shaoqin Shen, Zhinan Huang, Tong Yu, Zhuo Liu, Qihang Yang, et al. 2022. “Chromosome-level pepino genome provides insights into genome evolution and anthocyanin biosynthesis in Solanaceae.” *The Plant Journal* 110: 1128-1143. https://doi.org/10.1111/tpj.15728

2. Song, Xiaoming, Pengchuan Sun, Jiaqing Yuan, Ke Gong, Nan Li, Fanbo Meng, Zhikang Zhang, et al. 2021. “The celery genome sequence reveals sequential paleo-polyploidizations, karyotype evolution and resistance gene reduction in apiales.” *Plant Biotechnology Journal* 19: 731-744. [https://doi.org/10.1111/pbi.13499](https://doi.org/https://doi.org/10.1111/pbi.13499)

3. Marçais, Guillaume, Carl Kingsford. 2011. “A fast, lock-free approach for efficient parallel counting of occurrences of k-mers.” *Bioinformatics* 27: 764-770. <https://doi.org/10.1093/bioinformatics/btr011>

4. Cheng, Haoyu, Gregory T. Concepcion, Xiaowen Feng, Haowen Zhang, Heng Li. 2021. “Haplotype-resolved de novo assembly using phased assembly graphs with hifiasm.” *Nature Methods* 18: 170-175. <https://doi.org/10.1038/s41592-020-01056-5>

5. Servant, Nicolas, Nelle Varoquaux, Bryan R. Lajoie, Eric Viara, Chong-Jian Chen, Jean-Philippe Vert, Edith Heard, Job Dekker, Emmanuel Barillot. 2015. “HiC-Pro: an optimized and flexible pipeline for Hi-C data processing.” *Genome Biology* 16: 259. <https://doi.org/10.1186/s13059-015-0831-x>

6. Li, Heng, Richard Durbin. 2009. “Fast and accurate short read alignment with Burrows–Wheeler transform.” *Bioinformatics* 25: 1754-1760. <https://doi.org/10.1093/bioinformatics/btp324>

7. Zhang, Xingtan, Shengcheng Zhang, Qian Zhao, Ray Ming, Haibao Tang. 2019. “Assembly of allele-aware, chromosomal-scale autopolyploid genomes based on Hi-C data.” *Nature Plants* 5: 833-845. <https://doi.org/10.1038/s41477-019-0487-8>

8. Durand, Neva C., Muhammad S. Shamim, Ido Machol, Suhas S. P. Rao, Miriam H. Huntley, Eric S. Lander, Erez Lieberman Aiden. 2016. “Juicer Provides a One-Click System for Analyzing Loop-Resolution Hi-C Experiments.” *Cell Systems* 3: 95-98. https://doi.org/10.1016/j.cels.2016.07.002

9. Parra, Genis, Keith Bradnam, Ian Korf. 2007. “CEGMA: a pipeline to accurately annotate core genes in eukaryotic genomes.” *Bioinformatics* 23: 1061-1067. <https://doi.org/10.1093/bioinformatics/btm071>

10. Manni, Mosè, Matthew R Berkeley, Mathieu Seppey, Felipe A Simão, Evgeny M Zdobnov. 2021. “BUSCO Update: Novel and Streamlined Workflows along with Broader and Deeper Phylogenetic Coverage for Scoring of Eukaryotic, Prokaryotic, and Viral Genomes.” *Molecular Biology and Evolution* 38: 4647-4654. <https://doi.org/10.1093/molbev/msab199>

11. Ou, Shujun, Ning Jiang. 2017. “LTR_retriever: A Highly Accurate and Sensitive Program for Identification of Long Terminal Repeat Retrotransposons  ” *Plant Physiology* 176: 1410-1422. <https://doi.org/10.1104/pp.17.01310>

12. Hao, Zhaodong, Dekang Lv, Ying Ge, Jisen Shi, Dolf Weijers, Guangchuang Yu, Jinhui Chen. 2020. “RIdeogram: drawing SVG graphics to visualize and map genome-wide data on the idiograms.” *PeerJ Computer Science* 6: e251. <https://doi.org/10.7717/peerj-cs.251>

13. Flynn, Jullien M., Robert Hubley, Clément Goubert, Jeb Rosen, Andrew G. Clark, Cédric Feschotte, Arian F. Smit. 2020. “RepeatModeler2 for automated genomic discovery of transposable element families.” *Proceedings of the National Academy of Sciences* 117: 9451-9457. <https://doi.org/doi:10.1073/pnas.1921046117>

14. Bao, [Zhirong](https://pubmed.ncbi.nlm.nih.gov/?term=Bao+Z&cauthor_id=12176934), [Sean R](https://pubmed.ncbi.nlm.nih.gov/?term=Eddy+SR&cauthor_id=12176934) Eddy. 2002. “Automated de novo identification of repeat sequence families in sequenced genomes.” *Genome Research* 12: 1269-1276. <https://doi.org/10.1101/gr.88502>

15. Price, Alkes L., Neil C. Jones, Pavel A. Pevzner. 2005. “De novo identification of repeat families in large genomes.” *Bioinformatics* 21: i351-i358. <https://doi.org/10.1093/bioinformatics/bti1018>

16. Ou, Shujun, Ning Jiang. 2018. “LTR_retriever: A Highly Accurate and Sensitive Program for Identification of Long Terminal Repeat Retrotransposons  ” *Plant Physiology* 176: 1410-1422. <https://doi.org/10.1104/pp.17.01310>

17. Ellinghaus, David, Stefan Kurtz, Ute Willhoeft. 2008. “LTRharvest, an efficient and flexible software for de novo detection of LTR retrotransposons.” *BMC Bioinformatics* 9: 18. <https://doi.org/10.1186/1471-2105-9-18>

18. Xu, Zhao, Hao Wang. 2007. “LTR_FINDER: an efficient tool for the prediction of full-length LTR retrotransposons.” *Nucleic Acids Research* 35: W265-W268. <https://doi.org/10.1093/nar/gkm286>

19. Tarailo-Graovac, Maja, Nansheng Chen. 2009. “Using RepeatMasker to Identify Repetitive Elements in Genomic Sequences.” *Current Protocols in Bioinformatics* 25: 4.10.11-14.10.14. [https://doi.org/10.1002/0471250953.bi0410s25](https://doi.org/https://doi.org/10.1002/0471250953.bi0410s25)

20. Stanke, Mario, Burkhard Morgenstern. 2005. “AUGUSTUS: a web server for gene prediction in eukaryotes that allows user-defined constraints.” *Nucleic Acids Research* 33: W465-W467. <https://doi.org/10.1093/nar/gki458>

21. Korf, Ian. 2004. “Gene finding in novel genomes.” *BMC Bioinformatics* 5: 59. <https://doi.org/10.1186/1471-2105-5-59>

22. Keilwagen, Jens, Michael Wenk, Jessica L. Erickson, Martin H. Schattat, Jan Grau, Frank Hartung. 2016. “Using intron position conservation for homology-based gene prediction.” *Nucleic Acids Research* 44: e89-e89. <https://doi.org/10.1093/nar/gkw092>

23. Kim, Daehwan, Ben Langmead, Steven L. Salzberg. 2015. “HISAT: a fast spliced aligner with low memory requirements.” *Nature Methods* 12: 357-360. <https://doi.org/10.1038/nmeth.3317>

24. Pertea, Mihaela, Geo M. Pertea, Corina M. Antonescu, Tsung-Cheng Chang, Joshua T. Mendell, Steven L. Salzberg. 2015. “StringTie enables improved reconstruction of a transcriptome from RNA-seq reads.” *Nature Biotechnology* 33: 290-295. <https://doi.org/10.1038/nbt.3122>

25. Tang, Shiyuyun, Alexandre Lomsadze, Mark Borodovsky. 2015. “Identification of protein coding regions in RNA transcripts.” *Nucleic Acids Research* 43: e78-e78. <https://doi.org/10.1093/nar/gkv227>

26. Grabherr, Manfred G., Brian J. Haas, Moran Yassour, Joshua Z. Levin, Dawn A. Thompson, Ido Amit, Xian Adiconis, et al. 2011. “Full-length transcriptome assembly from RNA-Seq data without a reference genome.” *Nature Biotechnology* 29: 644-652. <https://doi.org/10.1038/nbt.1883>

27. Haas, Brian J., Arthur L. Delcher, Stephen M. Mount, Jennifer R. Wortman, Roger K. Smith Jr, Linda I. Hannick, Rama Maiti, et al. 2003. “Improving the Arabidopsis genome annotation using maximal transcript alignment assemblies.” *Nucleic Acids Research* 31: 5654-5666. <https://doi.org/10.1093/nar/gkg770>

28. Haas, Brian J., Steven L. Salzberg, Wei Zhu, Mihaela Pertea, Jonathan E. Allen, Joshua Orvis, Owen White, C. Robin Buell, Jennifer R. Wortman. 2008. “Automated eukaryotic gene structure annotation using EVidenceModeler and the Program to Assemble Spliced Alignments.” *Genome Biology* 9: R7. <https://doi.org/10.1186/gb-2008-9-1-r7>

29. Huerta-Cepas, Jaime, Damian Szklarczyk, Davide Heller, Ana Hernández-Plaza, Sofia K. Forslund, Helen Cook, Daniel R. Mende, et al. 2019. “eggNOG 5.0: a hierarchical, functionally and phylogenetically annotated orthology resource based on 5090 organisms and 2502 viruses.” *Nucleic Acids Research* 47: D309-D314. <https://doi.org/10.1093/nar/gky1085>

30. Mao, Xizeng, Tao Cai, John G. Olyarchuk, Liping Wei. 2005. “Automated genome annotation and pathway identification using the KEGG Orthology (KO) as a controlled vocabulary.” *Bioinformatics* 21: 3787-3793. <https://doi.org/10.1093/bioinformatics/bti430>

31. Boeckmann, Brigitte, Amos Bairoch, Rolf Apweiler, Marie-Claude Blatter, Anne Estreicher, Elisabeth Gasteiger, Maria J. Martin, et al. 2003. “The SWISS-PROT protein knowledgebase and its supplement TrEMBL in 2003.” *Nucleic Acids Research* 31: 365-370. <https://doi.org/10.1093/nar/gkg095>

32. Mistry, Jaina, Sara Chuguransky, Lowri Williams, Matloob Qureshi, Gustavo A Salazar, Erik L L Sonnhammer, Silvio C E Tosatto, et al. 2020. “Pfam: The protein families database in 2021.” *Nucleic Acids Research* 49: D412-D419. <https://doi.org/10.1093/nar/gkaa913>

33. Chan, Patricia P., Todd M. Lowe. 2019. tRNAscan-SE: Searching for tRNA Genes in Genomic Sequences. *Gene Prediction: Methods and Protocols* Springer New York, 1-14. <https://doi.org/10.1007/978-1-4939-9173-0_1>

34. Kalvari, Ioanna, Eric P Nawrocki, Nancy Ontiveros-Palacios, Joanna Argasinska, Kevin Lamkiewicz, Manja Marz, Sam Griffiths-Jones, et al. 2020. “Rfam 14: expanded coverage of metagenomic, viral and microRNA families.” *Nucleic Acids Research* 49: D192-D200. <https://doi.org/10.1093/nar/gkaa1047>

35. Nawrocki, Eric P., Sean R. Eddy. 2013. “Infernal 1.1: 100-fold faster RNA homology searches.” *Bioinformatics* 29: 2933-2935. <https://doi.org/10.1093/bioinformatics/btt509>

36. Li, Heng, Xiaowen Feng, Chong Chu. 2020. “The design and construction of reference pangenome graphs with minigraph.” *Genome Biology* 21: 265. <https://doi.org/10.1186/s13059-020-02168-z>

37. Mi, Huaiyu, Anushya Muruganujan, Dustin Ebert, Xiaosong Huang, Paul D Thomas. 2018. “PANTHER version 14: more genomes, a new PANTHER GO-slim and improvements in enrichment analysis tools.” *Nucleic Acids Research* 47: D419-D426. <https://doi.org/10.1093/nar/gky1038>

38. Yu, Guangchuang, Li-Gen Wang, Yanyan Han, Qing-Yu He. 2012. “clusterProfiler: an R package for comparing biological themes among gene clusters.” *Omics : a journal of integrative biology* 16: 284-287. <https://doi.org/10.1089/omi.2011.0118>

39. Marçais, Guillaume, Arthur L. Delcher, Adam M. Phillippy, Rachel Coston, Steven L. Salzberg, Aleksey Zimin. 2018. “MUMmer4: A fast and versatile genome alignment system.” *PLOS Computational Biology* 14: e1005944. <https://doi.org/10.1371/journal.pcbi.1005944>

40. Goel, Manish, Hequan Sun, Wen-Biao Jiao, Korbinian Schneeberger. 2019. “SyRI: finding genomic rearrangements and local sequence differences from whole-genome assemblies.” *Genome Biology* 20: 277. <https://doi.org/10.1186/s13059-019-1911-0>

41. Wang, Kai, Mingyao Li, Hakon Hakonarson. 2010. “ANNOVAR: functional annotation of genetic variants from high-throughput sequencing data.” *Nucleic Acids Research* 38: e164-e164. <https://doi.org/10.1093/nar/gkq603>

42. Katoh, Kazutaka, Daron M. Standley. 2013. “MAFFT Multiple Sequence Alignment Software Version 7: Improvements in Performance and Usability.” *Molecular Biology and Evolution* 30: 772-780. <https://doi.org/10.1093/molbev/mst010>

43. Rice, Peter, Ian Longden, Alan Bleasby. 2000. “EMBOSS: The European Molecular Biology Open Software Suite.” *Trends in Genetics* 16: 276-277. https://doi.org/10.1016/S0168-9525(00)02024-2

44. Ossowski, Stephan, Korbinian Schneeberger, José Ignacio Lucas-Lledó, Norman Warthmann, Richard M. Clark, Ruth G. Shaw, Detlef Weigel, Michael Lynch. 2010. “The Rate and Molecular Spectrum of Spontaneous Mutations in Arabidopsis thaliana.” *Science* 327: 92-94. <https://doi.org/10.1126/science.1180677>

45. Wang, Yupeng, Haibao Tang, Jeremy D. DeBarry, Xu Tan, Jingping Li, Xiyin Wang, Tae-ho Lee, et al. 2012. “MCScanX: a toolkit for detection and evolutionary analysis of gene synteny and collinearity.” *Nucleic Acids Research* 40: e49-e49. <https://doi.org/10.1093/nar/gkr1293>

46. Martin, Marcel. 2011. “Cutadapt removes adapter sequences from high-throughput sequencing reads.” *2011* 17: 3. <https://doi.org/10.14806/ej.17.1.200>

47. Robinson, Mark D., Davis J. McCarthy, Gordon K. Smyth. 2010. “edgeR: a Bioconductor package for differential expression analysis of digital gene expression data.” *Bioinformatics* 26: 139-140. <https://doi.org/10.1093/bioinformatics/btp616>

48. Kong, Lei, Yong Zhang, Zhi-Qiang Ye, Xiao-Qiao Liu, Shu-Qi Zhao, Liping Wei, Ge Gao. 2007. “CPC: assess the protein-coding potential of transcripts using sequence features and support vector machine.” *Nucleic Acids Research* 35: W345-W349. <https://doi.org/10.1093/nar/gkm391>

49. Sun, Liang, Haitao Luo, Dechao Bu, Guoguang Zhao, Kuntao Yu, Changhai Zhang, Yuanning Liu, Runsheng Chen, Yi Zhao. 2013. “Utilizing sequence intrinsic composition to classify protein-coding and long non-coding transcripts.” *Nucleic Acids Research* 41: e166-e166. <https://doi.org/10.1093/nar/gkt646>

50. Memczak, Sebastian, Marvin Jens, Antigoni Elefsinioti, Francesca Torti, Janna Krueger, Agnieszka Rybak, Luisa Maier, et al. 2013. “Circular RNAs are a large class of animal RNAs with regulatory potency.” *Nature* 495: 333-338. <https://doi.org/10.1038/nature11928>

51. Liu, Shiyi, Zitao Wang, Ronghui Zhu, Feiyan Wang, Yanxiang Cheng, Yeqiang Liu. 2021. Three Differential Expression Analysis Methods for RNA Sequencing: limma, EdgeR, DESeq2. *1940-087X* <https://doi.org/doi:10.3791/62528>

52. Kiełbasa, Szymon M., Nils Blï¿½thgen, Michael Fï¿½hling, Ralf Mrowka. 2010. “Targetfinder.org: a resource for systematic discovery of transcription factor target genes.” *Nucleic Acids Research* 38: W233-W238. <https://doi.org/10.1093/nar/gkq374>

53. Chen, Jiajia, Yuqing Zhong, Liangzhi Li. 2020. “miR-124 and miR-203 synergistically inactivate EMT pathway via coregulation of ZEB2 in clear cell renal cell carcinoma (ccRCC).” *Journal of Translational Medicine* 18: 69. <https://doi.org/10.1186/s12967-020-02242-x>

54. Ye, Tiantian, Xu Huang, Tianxiao Ma, Ying Li, Xiaofeng Wang, Hai Lu, Hua Xue. 2023. “Integrated Analysis of miRNAome and Transcriptome Identify Regulators of Elm Seed Aging.” *Plants* 12: 1719. <https://doi.org/10.3390/plants12081719>

55. Desfeux, Christine, Steven J. Clough, Andrew F. Bent. 2000. “Female Reproductive Tissues Are the Primary Target ofAgrobacterium-Mediated Transformation by the Arabidopsis Floral-Dip Method1.” *Plant Physiology* 123: 895-904. <https://doi.org/10.1104/pp.123.3.895>

56. Zhang, Zhijun, Chao Yang, Jing Xi, Yuting Wang, Jing Guo, Qianwei Liu, Yusong Liu, et al. 2024. “The MdHSC70–MdWRKY75 module mediates basal apple thermotolerance by regulating the expression of heat shock factor genes.” *The Plant Cell* 36: 3631-3653. <https://doi.org/10.1093/plcell/koae171>

**Figures S1-7**


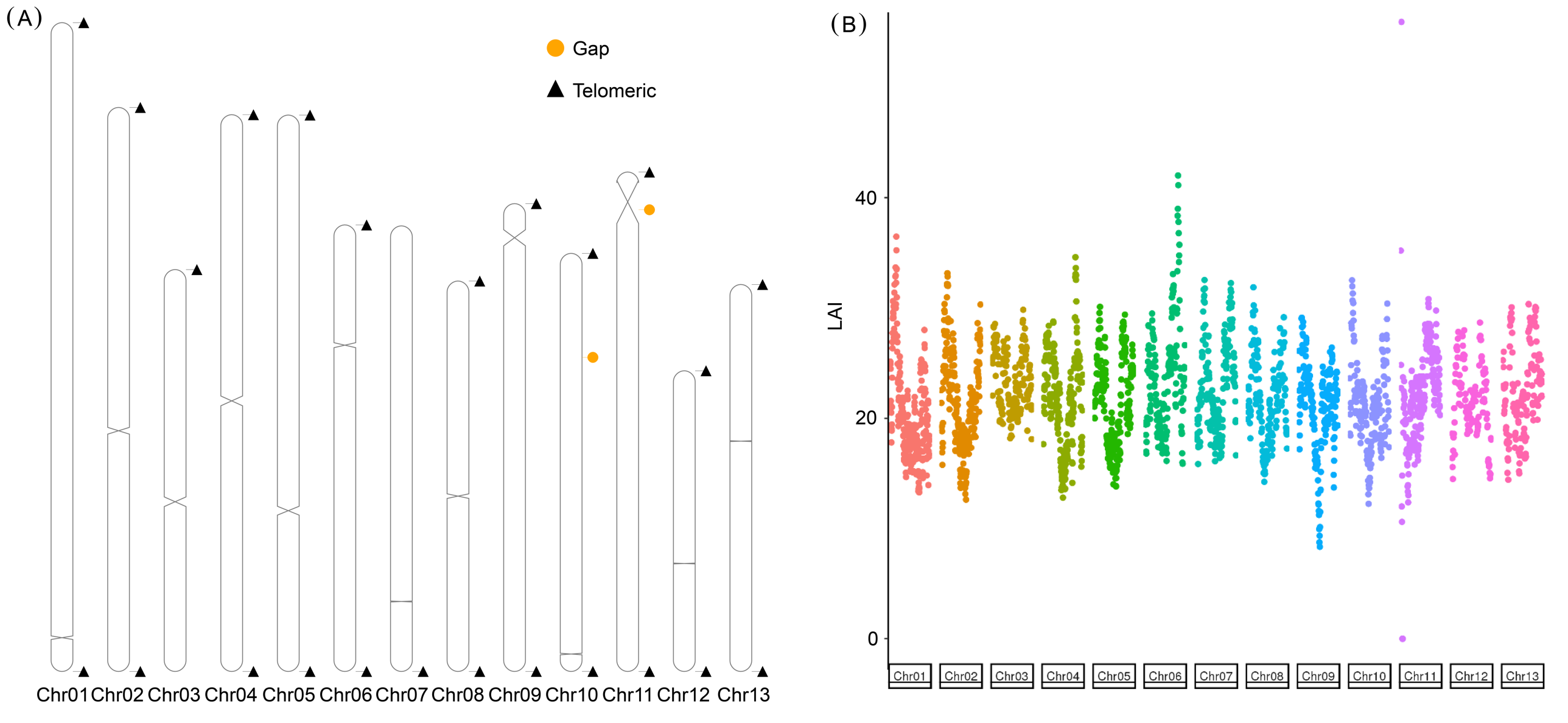


**Figure S1.** The assessment of Telomere-to-Telomere (T2T) genome of *R. liliiflorum*. (A) The telomeres, centromeres, and gap information in each chromosome. (B) The genome LTR assembly index (LAI) assessment of T2T genome.


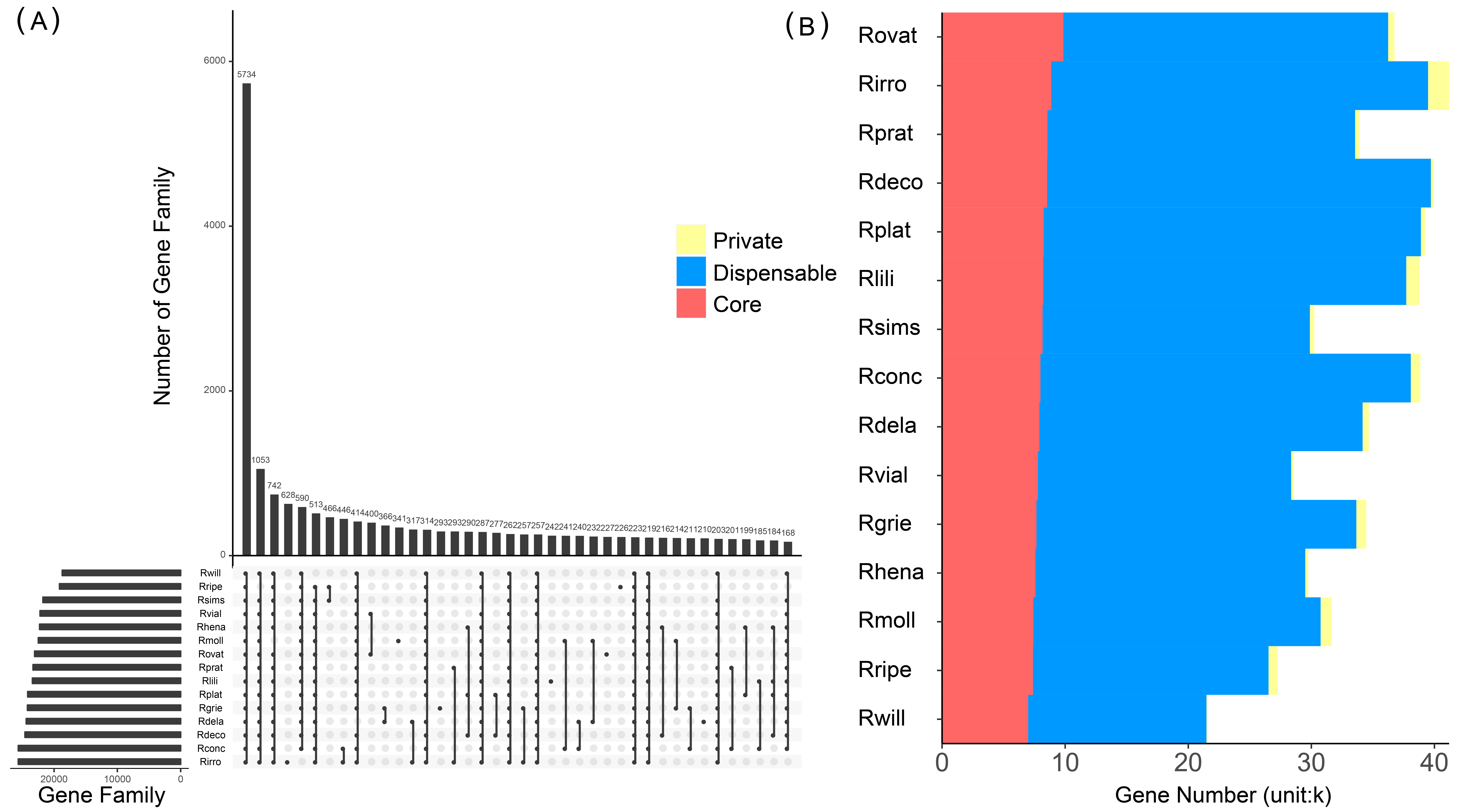


**Figure S2.** The gene family analysis of 15 species. (A) The total number of shared or specific gene families across the 15 species. (B) The number of genes in core gene families, dispensable gene families, and private gene families for each species.


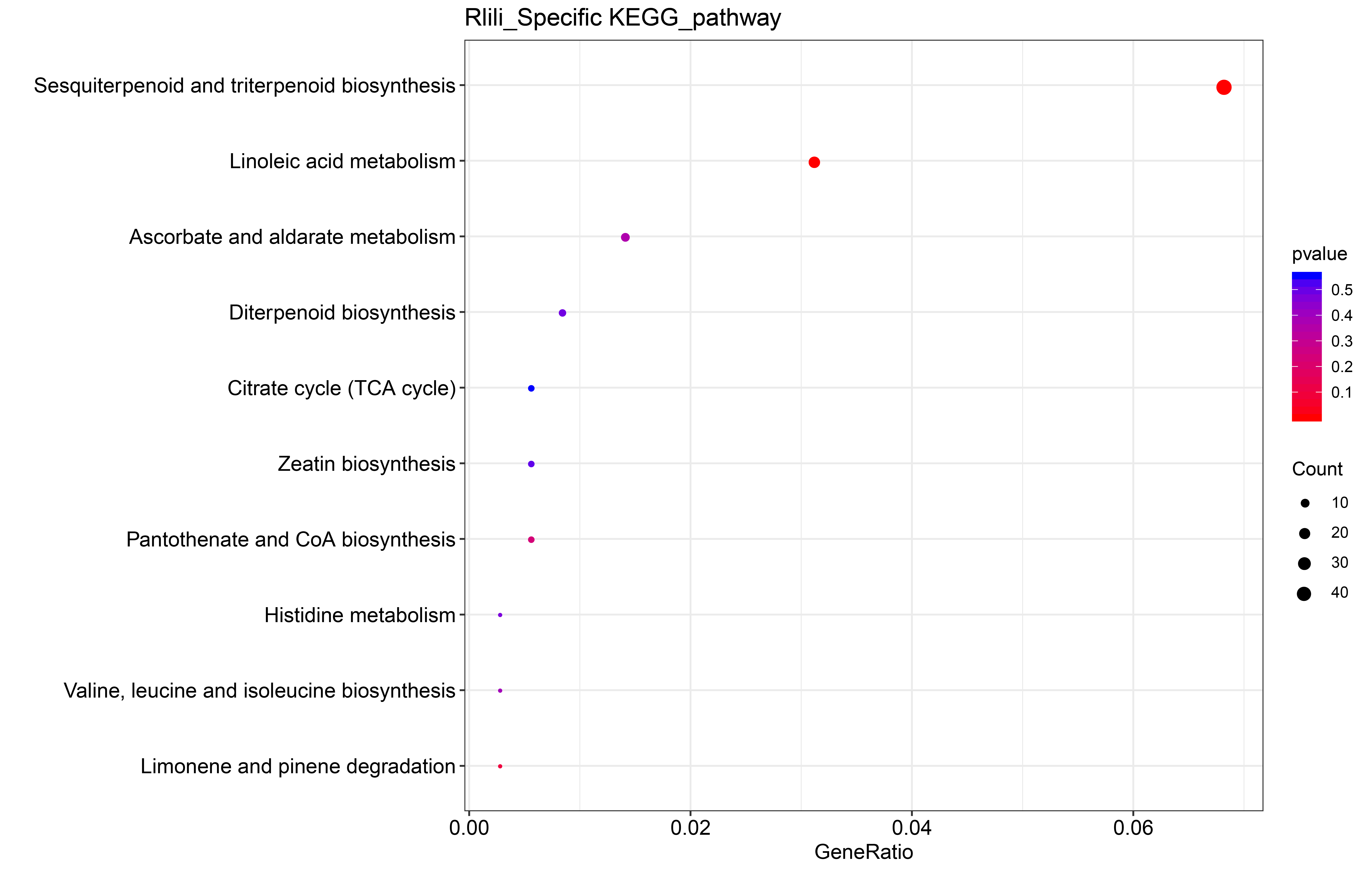


**Figure S3.** The KEGG functional enrichment analysis on all species-specific genes in *R. liliiflorum*.


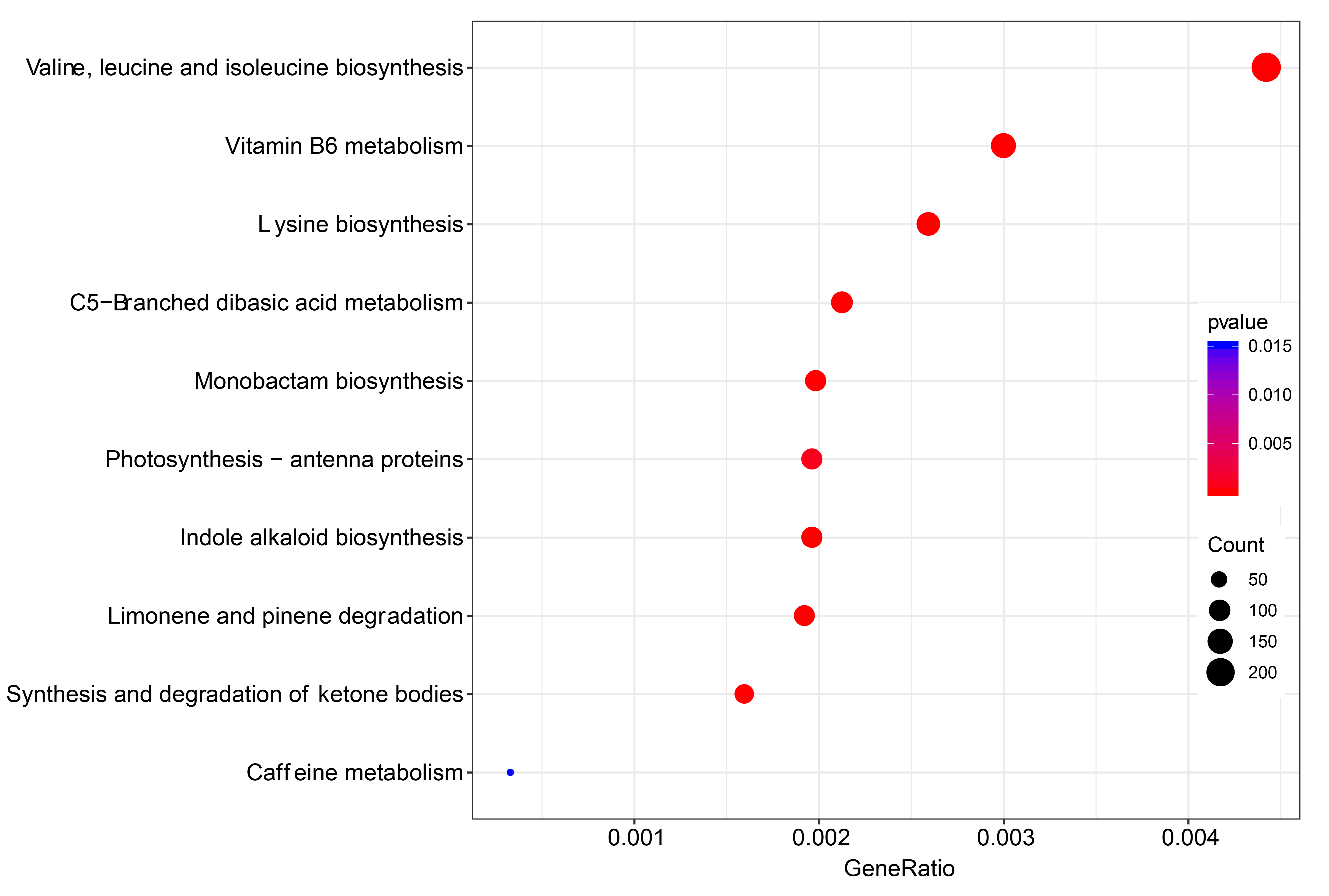


**Figure S4.** The KEGG functional enrichment analysis on all core cluster genes of 15 *Rhododendron* genomes.





**Figure S5.** The comparative genome visualization map shows the homology and rearrangement between each *Rhododendron* species and the reference T2T genome of *R. liliiflorum*.


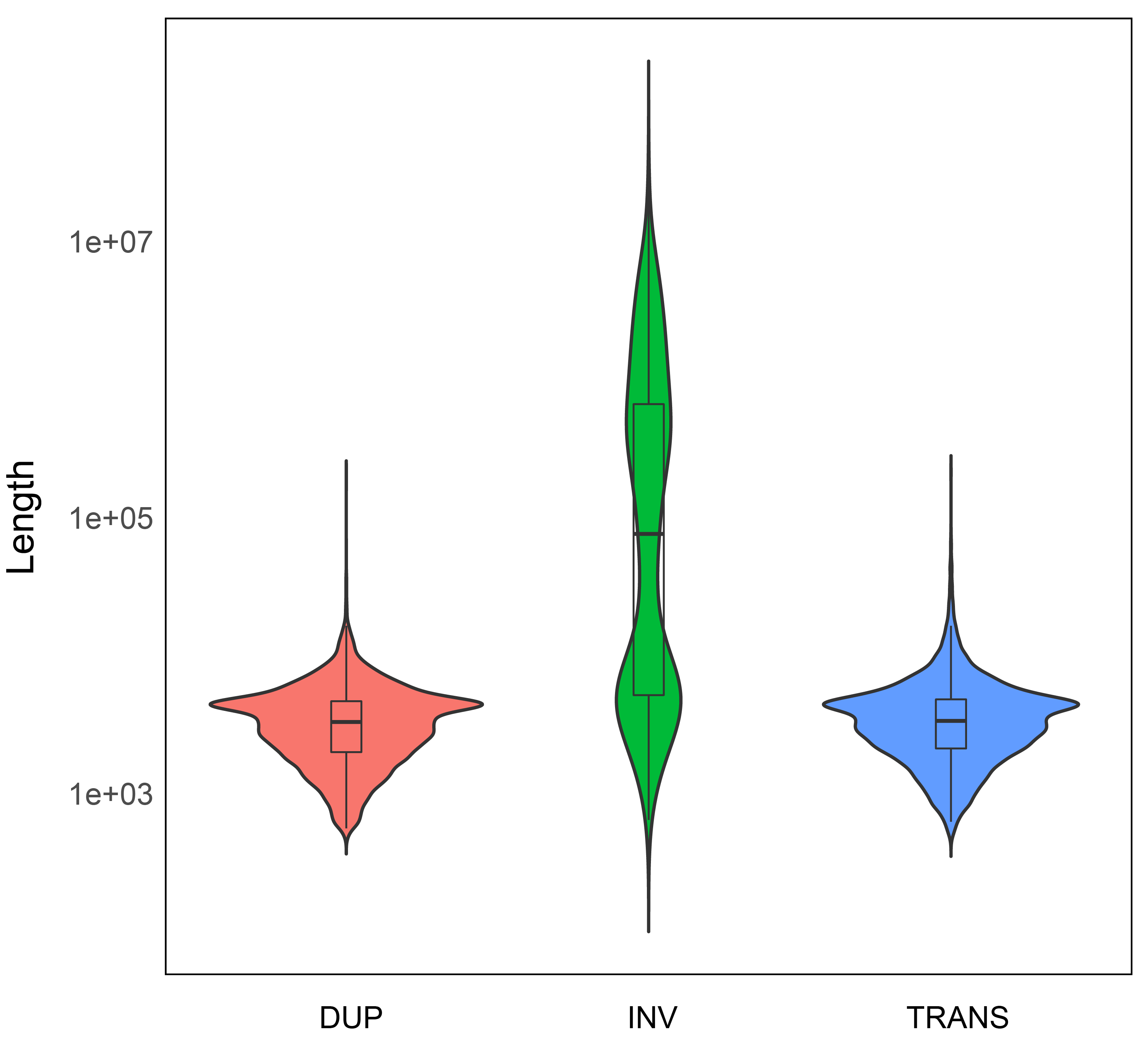


**Figure S6**. The length distribution of three structural variations (SVs) types, including duplication (DUP), inversion (INV), and translocation (TRANS) in the *Rhododendron* genome. The boxplot presents statistical information such as the median, quartiles, and extreme value ranges of the lengths of different types of structural variations.


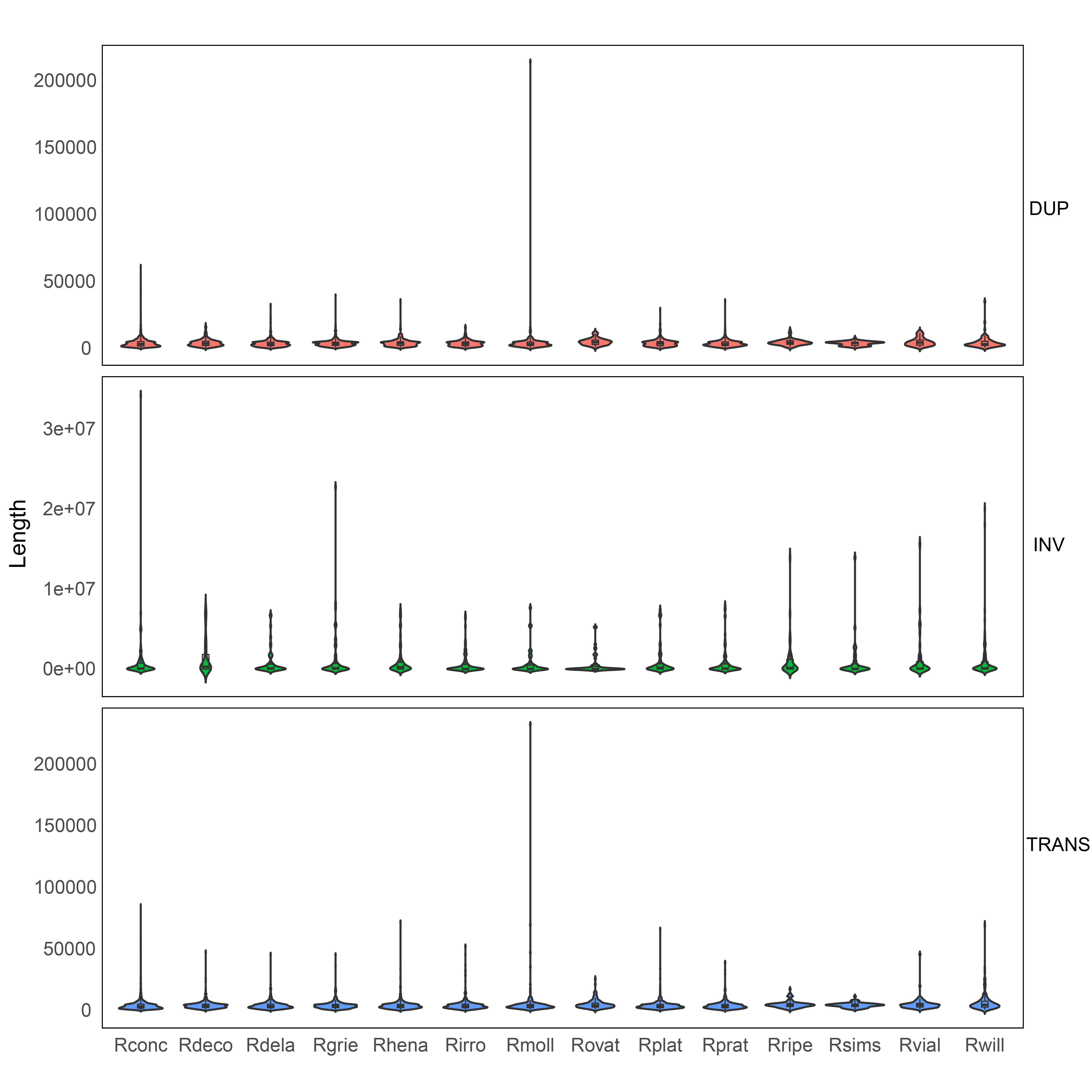


**Figure S7.** The length of duplication (DUP), translocation (TRANS), and inversion (INV) type of structural variations (SVs) in each *Rhododendron* species. The boxplot presents statistical information such as the median, quartiles, and extreme value ranges of the lengths of different types of structural variations.
